# The potential of multistress tolerant yeast, *Saccharomycodes ludwigii*, for second-generation bioethanol production

**DOI:** 10.1038/s41598-022-26686-x

**Published:** 2022-12-21

**Authors:** Warayutt Pilap, Sudarat Thanonkeo, Preekamol Klanrit, Pornthap Thanonkeo

**Affiliations:** 1 Department of Biotechnology, Faculty of Technology, Khon Kaen University, Khon Kaen, 40002 Thailand; 2 Walai Rukhavej Botanical Research Institute, Mahasarakham University, Maha Sarakham, 44150 Thailand; 3 Fermentation Research Center for Value Added Agricultural Products (FerVAAPs), Khon Kaen University, Khon Kaen, 40002 Thailand

**Keywords:** Biotechnology, Microbiology

## Abstract

Ethanol production at high temperatures using lignocellulosic biomass as feedstock requires a highly efficient thermo and lignocellulosic inhibitor-tolerant ethanologenic yeast. In this study, sixty-three yeast isolates were obtained from tropical acidic fruits using a selective acidified medium containing 80 mM glacial acetic acid. Twenty-nine of the yeast isolates exhibited significant thermo and acetic acid-tolerant fermentative abilities. All these isolates were classified into three major yeast species, namely *Saccharomycodes ludwigii*, *Pichia kudriavzevii*, and *P. manshurica*, based on molecular identification. *Saccharomycodes ludwigii* APRE2 displayed an ability to grow at high temperatures of up to 43 °C and exhibited significant multistress tolerance toward acetic acid, furfural, 5-hydroxymethyl furfural (5-HMF), and ethanol among the isolated yeast species. It can produce a maximum ethanol concentration of 63.07 g/L and productivity of 1.31 g/L.h in yeast extract malt extract (YM) medium containing 160 g/L glucose and supplemented with 80 mM acetic acid and 15 mM furfural as a cocktail inhibitor. When an acid-pretreated pineapple waste hydrolysate (PWH) containing approximately 106 g/L total sugars, 131 mM acetic acid, and 3.95 mM furfural was used as a feedstock, 38.02 g/L and 1.58 g/L.h of ethanol concentration and productivity, respectively, were achieved. Based on the results of the current study, the new thermo and acetic acid-tolerant yeast *S. ludwigii* APRE2 exhibited excellent potential for second-generation bioethanol production at high temperatures.

## Introduction

The world energy crisis and environmental pollution resulting from fossil fuel utilization have become one of the most significant global issues^1^. Approximately 88% of current energy utilization is derived from fossil fuels, such as crude oil, coal, and natural gas, and it is likely to be increased by 50% within the next 50 years. However, based on the current consumption rate, these fossil fuels may be depleted within approximately 120 years^2–5^. In addition, burning fossil fuels or improper disposal of fossil fuel wastes also causes severe environmental pollution, negatively impacting human and animal health^6^. Hence, a feasible way to investigate biofuel use as a cleaner alternative to fossil fuels is needed. Biofuels offer several benefits, such as enhanced long-term energy security, reduced emissions of greenhouse gases, reduced environmental pollution associated with fossil fuel utilization, and reduced demand for petroleum^5^. Many renewable biofuels have been made, such as bioethanol, biodiesel, biobutanol, and biohydrogen; among these, bioethanol is one of the most popular biofuels used worldwide due to its environmental sustainability and renewable nature compared to fossil fuels. Among the different generations of bioethanol production, the second-generation using lignocellulosic biomass as feedstock has received a great deal of interest over other generations of bioethanol production^7,8^. It not only has no impact on food security but also provides economic growth in rural areas^9,10^. Energy crops and green biomass residues or biowastes from industrial or agricultural sectors, such as sugarcane bagasse, cassava pulp, rice straw, wheat straw, corn cob, and pineapple wastes, are the primary feedstocks for second-generation bioethanol production^11^.

Lignocellulosic biomasses are typically composed of cellulose, hemicellulose, and lignin with a complex structure; thus, a pretreatment process to convert these lignocellulose materials into fermentable sugar monomers for growth and ethanol fermentation by ethanologenic microorganisms is needed. Several methods, such as physical, chemical, physico-chemical, and biological, have been used for lignocellulosic pretreatment^12^. Among the various techniques, the chemical pretreatment process, particularly a dilute acid pretreatment using sulfuric acid (H_2_SO_4_) at temperatures ranging from 120 °C to 180 °C, is the most commonly used because of its high efficiency in separating cell wall components, yielding high fermentable sugars, easy and low-cost operation due to the application of low-temperature conditions, and applicability to a wide range of biomasses^13–16^.

The lignocellulosic pretreatment process generates not only fermentable sugars but also lignocellulose-derived byproducts, which are known as lignocellulosic pretreatment inhibitors, that can inhibit microbial growth and metabolism as well as fermentation activity^17–20^. The principle lignocellulosic pretreatment inhibitors included weak acids (acetic acid and formic acid), furan derivatives (furfural and 5-hydroxymethyl furfural (5-HMF)), and phenolic compounds (phenol and O-methoxy phenol)^21,22^. Of these, acetic acid and furfural are two of the most predominant inhibitors in dilute acid-pretreated hydrolysate^23,24^. Acetic acid is formed by the deacetylation of hemicellulose and partly from cellulose and lignin degradation, while furfural is a common hemicellulose degradation product. The inhibitor content generated during the pretreatment process depends on the type of biomass, its concentration, and the pretreatment parameters, such as the time, operating temperature, and concentration of catalysts^21,25^. High concentrations of acetic acid (7.5–15.0 g/L) caused a considerable reduction in the maximum yeast cell biomass by prolonging the lag phase and reducing specific growth rates^26,27^. The dissociation of acetic acid inside the microbial cell decreases the intracellular pH, reducing cell viability and ethanol production performance^28^. Similarly, the microbial-specific growth rate, total cell mass, ATP yield, and ethanol production efficiency decreased as the furfural concentration in the fermentation medium increased^23^. Several strategies, such as the detoxification of the lignocellulosic hydrolysate using chemical or biological methods, an evolutionary adaptation of ethanologenic microorganisms in the presence of potent inhibitors, and microbial genetic engineering, have been performed to overcome the negative impact of these inhibitors^21,23^. However, these approaches are time-consuming, relatively high cost, and low efficiency. Therefore, a potential inhibitor-tolerant strain of ethanologenic microorganisms that can ferment hexose and pentose sugars in lignocellulosic hydrolysate is highly desirable for second-generation bioethanol production. Furthermore, the biological conversion process using cocktail enzymes for efficient sugar production and reduction of phenolic compounds could be an alternative to enhance microbial ethanol fermentation activity^22^. Several inhibitor-tolerant ethanologenic yeasts have been isolated and screened from different niches, and ethanol fermentation using lignocellulosic biomass as feedstocks has been reported, in which the most potential multistress-tolerant strains belonged to *Pichia* sp. For instance, Chamnipa et al.^29^ isolated *P. kudriavzevii* RZ8-1 from soil samples, and it exhibited an ability to withstand a high temperature of 45 °C and grow in a medium containing up to 7.5 g/L acetic acid and 12% (v/v) ethanol. This yeast strain can produce the highest ethanol concentrations of 35.51 and 33.84 g/L at 37 °C and 40 °C, respectively, using sugarcane bagasse hydrolysate as feedstock. *P. kudriavzevii* ITV-S42 isolated from sweet sorghum juice by Díaz-Nava et al.^30^ displayed significant potential for multistress tolerance, such as acetic acid (up to 24 g/L), furfural (up to 1 g/L) and 5-HMF (up to 1 g/L), and it produced the maximum ethanol yield and productivity of 0.376 g ethanol/g glucose and 0.54 g/L.h, respectively. Avchar et al.^31^ stated that *P. kudriavzevii* RGB3.2 isolated from buffalo rumen harbored significant tolerance capacity toward furfural, 5-HMF, acetic acid, and ethanol at concentrations up to 1.5 g/L, 3 g/L, 0.4% (v/v), and 10% (v/v), respectively. The yeast strain produced a maximum ethanol concentration of 9.1 g/L from rice straw hydrolysate using a simultaneous saccharification and fermentation (SSF) process at 45 °C. Phong et al.^32^ recently reported another multistress-tolerant yeast species, *Saccharomyces cerevisiae* HG1.1, isolated from soil samples in Vietnam. It exhibited significant tolerance toward acetic acid at 4 g/L and ethanol at 14% (v/v) and produced a maximum ethanol concentration of 36.85 g/L with a productivity of 3.07 g/L.h at 40 °C using pineapple waste hydrolysate (PWH) as feedstock.

To our knowledge, only a few studies have considered the isolation of thermotolerant yeast, which possesses multistress tolerance toward acetic acid and furfural from tropical acidic fruits. Thus, in this study, the isolation, screening, and selection of potential thermotolerant ethanologenic yeasts capable of multistress tolerance toward lignocellulosic inhibitors from tropical acidic fruits collected in northeastern Thailand were performed. The molecular identification, growth characterization, and ethanol production performance of the selected yeast isolates using glucose and PWH as feedstocks were also evaluated. The results from the present study demonstrated that the newly isolated thermo and acetic acid-tolerant yeast *S. ludwigii* APRE2 exhibited a high potential for second-generation bioethanol production from lignocellulosic biomass.

## Materials and methods

### Plant material

Tropical acidic fruits, namely pineapple (*Ananas comosus*), passion fruit (*Passiflora edulis*), Manila tamarind (*Pithecellobium dulce*), star fruit (*Averrhoa carambola*), ma fai or rambai fruit (*Baccaurea ramiflora*), dragon fruit (*Hylocereus undatus*), jujube (*Ziziphus jujuba*), korlan (*Nephelium hypoleucum*), mango (*Mangifera indica*), and orange (*Citrus* sp.), were used in this study. The plants used in this research are not wild but cultivated in Maha Sarakham, Roi Et, and Nong Khai provinces, Thailand. The pineapples and oranges were collected from local markets in Maha Sarakham province, while other fruits were collected from a local fruit garden in Roi Et province, Thailand, with the owner's permission, Mrs. Thongbol Punmill.

The pineapple waste (pineapple peels and core) used for ethanol fermentation was collected from the Food Services Center at Khon Kaen University with the permission of the University Office. A voucher specimen (dried material) was deposited at the Department of Biotechnology, Faculty of Technology, Khon Kaen University, with the code number KKUDB-PPC-2018–01. All methods were carried out following relevant guidelines in the method section.

### Sample collection and yeast isolation and screening

The collected tropical acidic fruits were washed and rinsed with sterilized distilled water to remove contaminants, including dust or microbes, on the fruit's surface and mashed into a homogeneous pulp using a grinder. Ten grams of fruit pulp was transferred into a 250-mL Erlenmeyer flask containing 100 mL of selective acidified yeast extract malt extract (YM) medium (3 g/L of yeast extract, 3 g/L of malt extract, 5 g/L of peptone, 10 g/L of glucose, and 80 mM glacial acetic acid) and incubated at 35 °C and 150 rpm for 3 days to obtain the thermotolerant yeast strains^33^. Following tenfold serial dilution, 10 μL of each sample was spread on high acetic acid YM agar supplemented with 80 mM glacial acetic acid and then incubated at 35 °C for 3 days. The different individualized colonies were selected based on their morphology, size, and color and then purified on YM agar medium. All the pure cultures were collected through repeated subculturing on YM agar medium incubated at 35 °C and maintained on a YM agar slant at 4 °C for short-term storage and in 50% (v/v) glycerol solution at − 20 °C for long-term storage.

The fermentative yeast strains were screened for their ability to ferment glucose using the Durham tube assay. The pure cultures of the yeast isolates were suspended in a 20 × 20 mm test tube with working volumes of 10 mL of YM medium (160 g/L glucose) supplemented with 80 mM glacial acetic acid and incubated at 35 °C and 150 rpm for 3 days. The yeast strains that could ferment sugar and accumulate a high level of CO_2_ in the Durham tube were selected for further experiments. All the experiments were performed in duplicate and repeated twice.

### Molecular identification of the selected fermentative yeasts

The selected yeast isolates were identified using the morphological and D1/D2 domain of large subunit (LSU) rDNA sequencing analysis^34^. The genomic DNA isolated from yeast cells was used as the template to amplify the D1/D2 domain of LSU-rDNA, which was performed using the specific primers NL-1 (5’-GCA TAT CAA TAA GCG GAG GAA AAG) and NL-4 (5’-GGT CCG TGT TTC AAG ACG G)^35^. Based on the manufacturer’s instructions, polymerase chain reaction (PCR) amplification was performed using a PCR master mix (QIAGEN, Dialunox GmbH, Germany). The resulting PCR products were separated by 0.8% agarose gel electrophoresis and purified using a NucleoSpin® Extract II Kit (Machery-Nagel GmbH & Co. KG, Germany) in accordance with the manufacturer’s instructions. The sequences of the D1/D2 domain of LSU-rDNA were analyzed using the dideoxy chain termination method, and homology searching analysis was performed using the FASTA and BLAST programs in the GenBank and DDBJ databases.

Phylogenetic analysis was constructed using the MEGA XI program^36^, and the tree topologies were analyzed by bootstrapping with 1000 replicates based on the neighbor-joining method^37^.

### Growth characterization and sugar assimilation of selected fermentative yeasts

The growth ability of the isolated yeasts was tested under high-temperature conditions by transferring the pure yeast isolate cultures into a 20 × 20 mm test tube containing 10 mL of YM medium supplemented with 80 mM glacial acetic acid and incubated at different temperatures of 30 °C, 37 °C, 40 °C, 43 °C, and 45 °C in a controlled temperature incubator shaker (JSSI-100 T, JS Research Inc., Korea) at 150 rpm for 48 h. The growth was monitored in terms of cell viability using a haemacytometer (H-0004, Boeco, Germany) with methylene blue staining. The sugar assimilation profile of the isolated yeasts was evaluated by inoculating the yeast cells into a YM medium supplemented with 20 g/L of different sugars, namely glucose, galactose, xylose, arabinose, maltose, sucrose, and lactose. After incubation at 30 °C for 48 h, the sugar assimilation was determined by following the standard protocol^38^.

### Inhibitory effect of lignocellulosic pretreatment inhibitors on the growth of selected fermentative yeasts

The inhibitory effect of lignocellulosic pretreatment inhibitors, including acetic acid, furfural, 5-HMF, and ethanol, on isolated yeast growth was evaluated using a liquid culture assay. The yeast inoculum was prepared by transferring a loopful of pure culture of isolated yeasts into a 125-mL Erlenmeyer flask containing 50 mL of YM medium, which was incubated at 35 °C and 150 rpm for 18 h (when the growth reached the mid-exponential phase). Cells were collected by centrifugation at 1844 × g, resuspended in sterile 0.85% NaCl solution to obtain an initial cell concentration of 1 × 10^6^ cells/mL, and used as a starter culture for further experiments^39^. For acetic acid, furfural, 5-HMF, and ethanol stresses, cells were transferred into a 250-mL Erlenmeyer flask containing 100 mL of YM medium supplemented with different concentrations of acetic acid (80 mM, 120 mM, and 160 mM), furfural (5 mM, 10 mM, and 15 mM), 5-HMF (10 mM, 15 mM, and 20 mM), or ethanol (4%, 6%, and 8% v/v)^33,40^ and then incubated at 35 °C in a controlled temperature incubator shaker (JSSI-100 T, JS Research Inc., Korea) at 150 rpm. YM medium without inhibitor supplementation was used as the control. After 48 h of incubation, the viability of the yeast cells was measured using a haemacytometer (H-0004, Boeco, Germany) with methylene blue staining.

### Effect of acetic acid and furfural on ethanol production by selected fermentative yeasts

Acetic acid and furfural are the predominant lignocellulosic pretreatment inhibitors that negatively affect microbial growth and ethanol production potential^23,24^. In this study, the effect of acetic acid and furfural on the ethanol production capability of the selected yeasts was evaluated using a YM medium containing 160 g/L glucose supplemented with 80 mM acetic acid or 15 mM furfural as a fermentation medium. YM medium containing 160 g/L glucose without inhibitor supplementation was used as a control treatment. A starter culture of each isolated yeast was transferred into a fermentation medium with an initial cell concentration of 1 × 10^7^ cells/mL and incubated at 37 °C in a controlled temperature incubator shaker (JSSI-100 T, JS Research Inc., Korea) at 150 rpm. Fermentation broths were collected at specific time intervals, and the ethanol concentration and productivity were analyzed.

### Ethanol production efficiency of selected yeasts under multistress conditions using inhibitor cocktail

The effect of multistress conditions on ethanol production by selected isolated yeasts was investigated using YM medium containing 160 g/L glucose and supplemented with an inhibitor cocktail comprising 80 mM acetic acid and 15 mM furfural as a fermentation medium. A starter culture of selected yeast isolates was transferred into a 250-mL Erlenmeyer flask containing 100 mL fermentation medium with an initial cell concentration of 1 × 10^7^ cells/mL. The flasks were incubated at 37 °C in a controlled temperature incubator shaker (JSSI-100 T, JS Research Inc., Korea) at 150 rpm, and samples were collected at specific time intervals during ethanol fermentation and then analyzed. A fermentation medium without any inhibitors was used as a control treatment.

### Ethanol production from pineapple waste hydrolysate (PWH) by selected yeast

PWH was prepared using the method described by Rattanapoltee and Kaewkannetra^41^. In brief, 0.5 × 0.5 cm dried pineapple wastes were treated with 0.5% (v/v) sulfuric acid (H_2_SO_4_) at 121 °C for 15 min using an autoclave (HV-50II, Hirayama, Japan). After acid hydrolysis, the pellet was removed by centrifugation, and the resulting supernatant was collected and used as feedstock for ethanol fermentation. The PWH was stored at − 20 °C until use. The chemical compositions in the PWH, including sugars, acetic acid, furfural, 5-HMF, and phenolic acid, were analyzed using high-performance liquid chromatography (HPLC) with a refractive index (RI) detector (Shimadzu, Kyoto, Japan) using Aminex HPX-87H column (300 × 7.8 mm) (Bio-Rad, Hercules, CA, USA) at 50 °C. 5 mM H_2_SO_4_ was used as mobile phase at a flow rate of 0.6 ml/min.

The batch ethanol fermentation was performed using a 250-mL Erlenmeyer flask containing 100 mL of PWH as a fermentation medium. The medium was inoculated with selected yeast cells with an initial cell concentration of 1 × 10^7^ cells/mL and incubated at 37 °C in a controlled temperature incubator shaker (JSSI-100 T, JS Research Inc., Korea) at 150 rpm. The samples were collected at specific time intervals during ethanol fermentation and analyzed.

### Analytical methods

The total residual sugars in the fermentation broth were measured using the phenol sulfuric acid method^42^. The direct counting method was employed using a haemacytometer (H-0004, Boeco, Germany) with methylene blue staining to determine the viable yeast cell number^43^. The ethanol concentration was determined by gas chromatography (GC) (Shimadzu GC-14B, Kyoto, Japan) using a polyethylene glycol (PEG-20 M)-packed column with a flame ionization detector, following the protocol described by Laopaiboon et al.^44^. The ethanol yield (*Yp/s*, g/g), volumetric ethanol productivity (*Qp*, g/L.h), and conversion efficiency or yield efficiency (*Ey*, %) were calculated as described by Nuanpeng et al.^45^.

All the experiments were performed in duplicate and repeated twice. The results were expressed as the means ± standard deviation (SD), and the mean differences between each treatment were analyzed by Duncan's multiple range test (DMRT) at a probability of *p* ≤ 0.05 using the SPSS program for Windows.

## Results and discussion

### Isolation and screening of fermentative yeasts

High-temperature ethanol fermentation (HTEF) using highly efficient thermo and acetic acid-tolerant ethanologenic yeasts is a promising strategy for high-temperature second-generation bioethanol production using lignocellulosic biomass as feedstock. Based on the literature review, only a few reports regarding the isolation of thermo and acetic acid-tolerant yeasts from tropical acidic fruits. Thus, this study exploited a new potential source for isolation, screening, and selecting high-potential thermo and acetic acid-tolerant yeasts for high-temperature ethanol production. Based on the morphological characteristics of the yeast, a total of sixty-three yeast isolates were obtained from different tropical acidic fruit samples. Most yeast colonies were circular with a diameter of 1–2 mm, but their colors varied from white to cream or tan (data not shown). The ethanologenic fermentative yeasts could be preliminarily screened using the Durham tube assay, one of the widely used techniques for screening and selecting high-potential thermotolerant ethanol-producing yeasts^45,46^. Based on the Durham tube assay at 35 °C using YM medium containing 160 g/L glucose supplemented with 80 mM glacial acetic acid, twenty-nine yeast isolates, or 46% of the yeast isolates, exhibited a high capacity to ferment glucose and accumulate high levels of CO_2_ in the tubes. Thus, all twenty-nine fermentative yeast isolates were selected for further characterization.

Several ethanologenic fermentative yeasts have been isolated from different sources, for instance, soil, alcoholic beverages, plant materials, such as the leaves and stalks of Jerusalem artichoke, sugarcane juice, sweet sorghum juice, rotten fruits, and plant flowers^29,45–48^, buffalo rumen^31^ and naturally fermented foods^49^. Based on the results obtained in the current study, tropical acidic fruits are also a potential niche for the isolation of ethanologenic fermentative yeasts, among other sources.

### Molecular identification of selected fermentative yeasts

**Figure 1:**
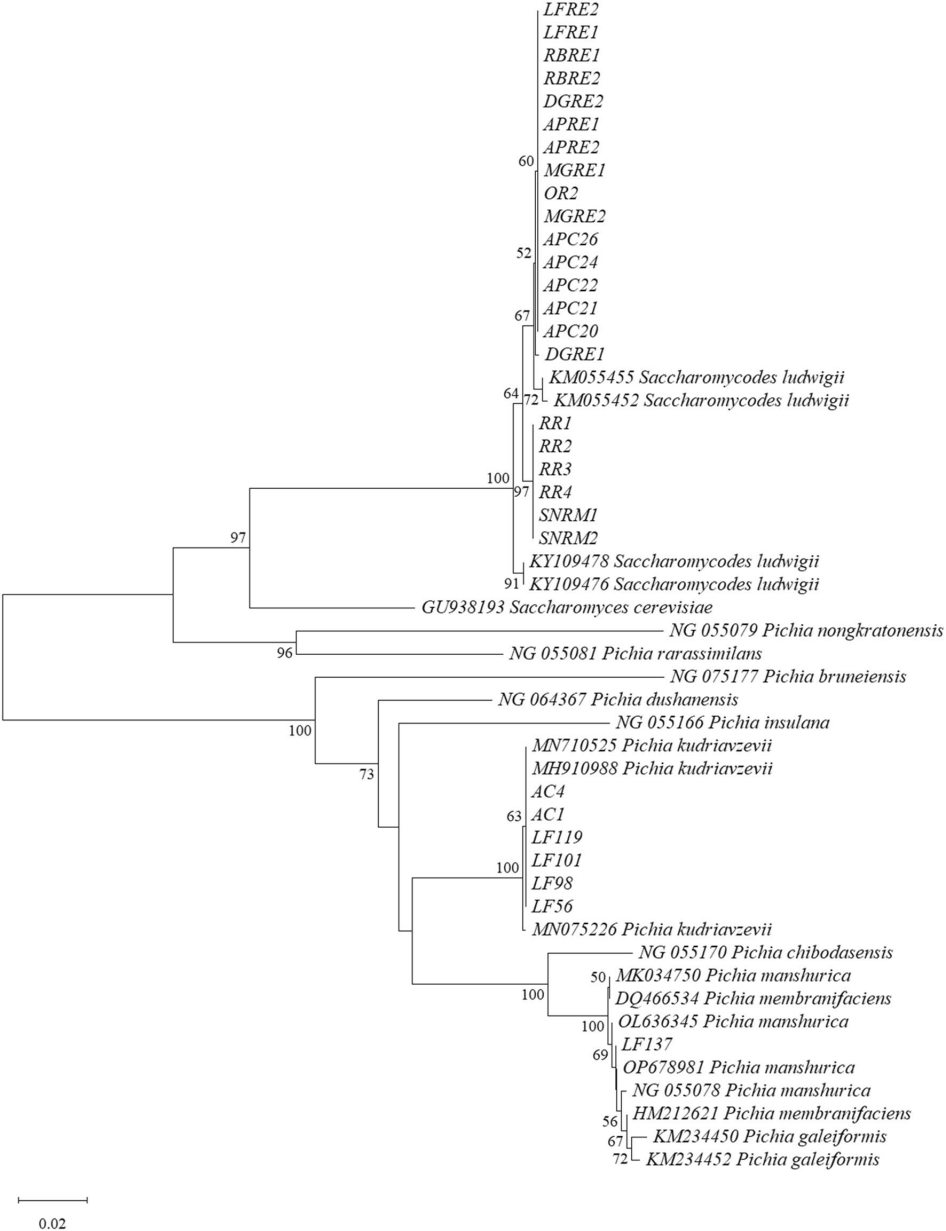
Phylogenetic tree of the D1/D2 domain of LSU rDNA gene from neighbor-joining depicting relationships among type strains of selected yeast species. Numbers at the nodes indicate the percentage bootstrap values based on 1000 replications.

The twenty-nine selected yeast isolates were identified at the species level by sequencing analysis of the D1/D2 domain of LSU-rDNA. Based on the homology and phylogenetic analyses of the nucleotide sequences in the GenBank database (https://www.ncbi.nlm.nih.gov/genbank/), three major yeast species were clarified, i.e., *Saccharomycodes ludwigii*, *Pichia kudriavzevii* (former name: *Issatchenkia orientalis*), and *P. manshurica* (*P. galeiformis)* (Fig. 1). *Saccharomycodes ludwigii* was the most common yeast species among the selected fermentative yeasts. Twenty-two isolates, namely, ACP20, ACP21, ACP22, ACP24, ACP26, RBRE1, RBRE2, DGRE1, DGRE2, LFRE1, LFRE2, SNRM1, SNRM2, APRE1, APRE2, MGRE1, MGRE2, RR1, RR2, RR3, RR4, and OR2, were identified as *S.* *ludwigii* with 99.3–100% sequence identity. Six isolates, LF56, LF98, LF101, LF119, AC1, and AC4, belonged to *P. kudriavzevii* with 100% sequence identity, and one isolate, i.e., LF137, was identified as *P. manshurica* with 99.5% sequence identity (Table 1). Interestingly, *S.* *ludwigii* was primarily found in almost all the fruit samples except passion fruit, Manila tamarind, and korlan, while *P. manshurica* was restricted to star fruit only. *P. manshurica* was first isolated from an alcoholic drink in Manchuria^50^, but it was later found in other different sources, e.g., waste sediments and sugarcane juice from sugarcane factories^51^, soil^48^, and several fermented foods and beverages, such as table olives, cocoa beans, koumiss, vinegar, fermented tea or kombucha, and spoiled wines^52–54^. Although it is considered one of the spoilage yeasts that cause severe problems for wine industries, *P. manshurica* also has good potential for ethanol and vinegar production^51,55^.

**Table 1:** Selected ethanologenic fermentative yeasts isolated from tropical acidic fruits and their molecular identification.

Sources	Yeast isolates	Yeast species	% identity	GenBank Accession number
Pineapple (*Ananas comosus*)	ACP20	*Saccharomycodes ludwigii*	99.5	OP678949
	ACP21	*S. ludwigii*	99.5	OP678950
	ACP22	*S. ludwigii*	99.5	OP678951
	ACP24	*S. ludwigii*	99.5	OP678952
	ACP26	*S. ludwigii*	99.5	OP678953
	RR1	*S. ludwigii*	99.3	OP678954
	RR2	*S. ludwigii*	99.3	OP678955
	RR3	*S. ludwigii*	99.3	OP678956
	RR4	*S. ludwigii*	99.3	OP678957
Passion fruit (*Passiflora edulis*)	LF56	*Pichia kudriavzevii*	100	OP678975
Manila tamarind (*Pithecellobium dulce*)	LF98	*P. kudriavzevii*	100	OP678976
	LF101	*P. kudriavzevii*	100	OP678977
Star fruit (*Averrhoa carambola*)	LF119	*P. kudriavzevii*	100	OP678978
	LF137	*P. manshurica*	99.5	OP678981
	LFRE1	*S. ludwigii*	99.5	OP678958
	LFRE2	*S. ludwigii*	99.5	OP678959
	SNRM1	*S. ludwigii*	99.3	OP678960
	SNRM2	*S. ludwigii*	99.3	OP678961
Ma Fai (*Baccaurea ramiflora*)	RBRE1	*S. ludwigii*	99.5	OP678962
	RBRE2	*S. ludwigii*	99.5	OP678963
Dragon fruit (*Hylocercus undatus*)	DGRE1	*S. ludwigii*	100	OP678964
	DGRE2	*S. ludwigii*	99.5	OP678965
Jujube (*Ziziphus jujuba*)	APRE1	*S. ludwigii*	99.5	OP678966
	APRE2	*S. ludwigii*	99.5	OP678967
Korlan (*Nephelium hypoleucum*)	AC1	*P. kudriavzevii*	100	OP678979
Mango (*Mangifera indica*)	AC4	*P. kudriavzevii*	100	OP678980
	MGRE1	*S. ludwigii*	99.5	OP678968
	MGRE2	*S. ludwigii*	99.5	OP678969
Orange (*Citrus* sp.)	OR2	*S. ludwigii*	99.5	OP678970

The common thermotolerant fermentative yeast, *P. kudriavzevii*, is widely distributed in natural habitats. It has been isolated from several niches, such as soil, fruits, sawdust^29,47,48^, waste sediments, and sugarcane juice from sugarcane factories^51^, buffalo rumen^31^, and naturally fermented foods^49^. It is also known as a typical multistress-tolerant yeast, including thermal, ethanol, furfural, 5-HMF, acetic acid, and osmotic stress^31,38,49^, exhibiting a high potential to produce ethanol^29,48^, single-cell protein^56^, and D-xylonate^57^.

*Saccharomycodes ludwigii*, a budding yeast belonging to the *Saccharomycodeacea* family, is also known as a spoilage yeast in winemaking due to its high tolerance to sulfur dioxide (SO_2_)^58^. This nonconventional yeast has been isolated from a variety of niches, such as sweet wines^59^, soil^60^, insects^61^, and coconut inflorescence sap^62^. It exhibits a high potential for biofuel and beverage production, especially ethanol, wine, and low-alcohol beer^63^, as well as other chemical production, such as ethyl acetate^48^, succinic acid, and glycerol^64,65^.

It should be noted in the current study that the traditionally used yeast for ethanol production, *Saccharomyces cerevisiae*, and the common thermotolerant fermentative yeast, *Kluyveromyces marxinus*, were not isolated in recent research, which might be due to the synergetic effect of relatively high temperature and acetic acid stress. Although some strains of *S. cerevisiae* and *K. marxianus* can withstand high temperature and acetic acid conditions, most are sensitive to both stresses’ synergetic effects.

### Growth characterization and sugar assimilation of selected fermentative yeasts

The growth performance under thermal stress or the thermotolerance ability of the selected ethanologenic fermentative yeasts was evaluated by culturing the yeast cells in YM medium supplemented with 80 mM glacial acetic acid and incubated at 30 °C (control), 37 °C, 40 °C, 43 °C, and 45 °C for 48 h. As shown in Table 2, all the yeast isolates grew well at 37 °C with slightly lower cell viability than that at 30 °C. Interestingly, *S. lugwigii* ACP22 showed 100% cell viability, the same as the control treatment at 30 °C. All the selected yeast isolates showed good growth at 40 °C, with cell viability of 42.15–78.98% compared to a control treatment at 30 °C. Although all of the selected yeast isolates could grow at 43 °C, their cell viabilities were markedly decreased to 5.80–14.80%, 25.36–36.20%, and 24.46% for *S. ludwigii*, *P. kudriavzevii*, and *P. manshurica*, respectively, compared with the control treatment. No growth was observed for *S. ludwigii* at 45 °C, while *P. kudriavzevii* and *P. manshurica* exhibited a low level of growth at this temperature, suggesting that *P. kudriavzevii* and *P. manshurica* were more thermally resistant than *S. ludwigii*. Based on the growth capacity of all selected yeast isolates at temperatures higher than 40 °C, they were considered thermotolerant yeasts^66^.

**Table 2:** Relative cell viability (%) under different temperatures and the sugar assimilation of the selected ethanologenic fermentative yeasts.

Yeast isolates	Temperatures (°C)^a^					Sugar assimilation^b^						
	30	37	40	43	45	Glu	Gal	Xyl	Ara	Mal	Suc	Lac
ACP20	100	95.23	63.42	14.80	0.00	+ +	−	−	−	−	+ +	−
ACP21	100	85.82	53.94	9.53	0.00	+ +	−	−	−	−	+ +	−
ACP22	100	100.00	68.15	12.46	0.00	+ +	−	−	−	−	+ +	−
ACP24	100	98.54	60.33	10.03	0.00	+ +	−	−	−	−	+ +	−
ACP26	100	80.98	50.41	8.24	0.00	+ +	−	−	−	−	+ +	−
RR1	100	95.01	60.22	11.30	0.00	+ +	−	−	−	−	+ +	−
RR2	100	96.01	58.93	9.61	0.00	+ +	−	−	−	−	+ +	−
RR3	100	94.02	54.21	7.44	0.00	+ +	−	−	−	−	+ +	−
RR4	100	94.40	50.89	8.90	0.00	+ +	−	−	−	−	+ +	−
LF56	100	95.20	76.22	35.22	1.46	+ +	−	+	−	−	+	−
LF98	100	96.64	78.98	36.20	0.75	+ +	−	+	−	−	+	−
LF101	100	90.50	70.12	27.93	1.63	+ +	−	+	−	−	+	−
LF119	100	86.23	65.25	30.45	3.87	+ +	−	+	−	−	+	−
LF137	100	90.05	69.71	24.46	0.59	+ +	−	+	−	−	+	−
LFRE1	100	80.37	50.30	10.74	0.00	+ +	−	−	−	−	+ +	−
LFRE2	100	79.63	48.19	12.03	0.00	+ +	−	−	−	−	+ +	−
SNRM1	100	87.93	58.06	14.80	0.00	+ +	−	−	−	−	+ +	−
SNRM2	100	85.96	51.42	9.07	0.00	+ +	−	−	−	−	+ +	−
RBRE1	100	78.34	45.37	7.35	0.00	+ +	−	−	−	−	+ +	−
RBRE2	100	80.65	46.72	9.21	0.00	+ +	−	−	−	−	+ +	−
DGRE1	100	82.13	48.73	10.33	0.00	+ +	−	−	−	−	+ +	−
DGRE2	100	82.83	50.41	12.84	0.00	+ +	−	−	−	−	+ +	−
APRE1	100	79.82	42.15	5.80	0.00	+ +	−	−	−	−	+ +	−
APRE2	100	75.54	46.62	9.13	0.00	+ +	−	−	−	−	+ +	−
AC1	100	94.33	78.35	28.03	0.74	+ +	−	+	−	−	+	−
AC4	100	90.54	73.21	25.36	1.37	+ +	−	+	−	−	+	−
MGRE1	100	85.54	52.29	11.03	0.00	+ +	−	−	−	−	+ +	−
MGRE2	100	87.22	50.21	8.23	0.00	+ +	−	−	−	−	+ +	−
OR2	100	93.10	57.39	13.45	0.00	+ +	−	−	−	−	+ +	−

^a^Relative cell viability compared to control treatment (30 °C).

^b^Glu, glucose; Gal, galactose; Xyl, xylose; Ara, arabinose; Mal, maltose; Suc, sucrose; Lac, lactose; (+ +) good growth; ( +) moderate growth; and ( −) no growth.

The ability of yeast cells to use a wide range of sugars, especially fermentable sugars liberated from lignocellulosic materials after the pretreatment and hydrolysis process, is a promising approach for second-generation bioethanol production. In the present study, the utilization of the main sugars from lignocellulosic materials, including glucose, galactose, xylose, and arabinose, and the disaccharides primarily found in fruit wastes or dairy processes, such as maltose, sucrose, and lactose, was determined, and the results are summarized in Table 2. All of the selected yeast isolates can use glucose and sucrose as carbon sources. Additionally, a group of *P. kudriavzevii* strains (LF56, LF98, LF101, LF119, AC1, and AC4) and a *P. manshurica* strain (LF137) can also use xylose as a carbon source, similar to studies reported by Avchar et al.^31^ and Aouine et al.^49^. However, there were no selected yeast isolates using galactose, arabinose, maltose, and lactose as carbon sources in this study. The ability to use galactose, arabinose and maltose as carbon sources has been reported in *P. kudriavzevii* strains RGB7.2, RGB7.7, RGB8.5^31^, YSR1, and YSR29^49^. Notably, the sugar utilization profiles of the newly isolated yeast *S. ludwigii* in this study were in accordance with the findings of De Francesco et al.^67^.

### Inhibitory effect of lignocellulosic pretreatment inhibitors on the growth of selected fermentative yeasts

The pretreatment of lignocellulosic biomasses releases not only fermentable sugar monomers but also other byproducts known as lignocellulosic pretreatment inhibitors. Many are known to be cytotoxic, which causes severe effects on microbial growth and fermentation activity. The majority of these lignocellulosic pretreatment inhibitors included aliphatic acids, especially acetic acid and formic acid, which originate from the hemicellulose fraction and partly from cellulose and lignin degradation, phenolic compounds that are derived from lignin polymers, and furan derivatives, such as furfural and 5-HMF, which are generated during the dehydration reactions of pentose and hexose sugars, respectively^21,68^. The concentrations of these inhibitory compounds strongly depend on the types of feedstock and the pretreatment processes^21^. These inhibitory compounds are typically fully or partially removed via different detoxification processes, which are costly and complicated, such as physical, chemical, and biological, before being used for ethanol or other bioproduct formation^69^. Applying potential ethanologenic yeasts that are tolerant to lignocellulosic inhibitors is a promising and cost-effective technique for lignocellulosic ethanol production.

It has previously been reported that the damage induced by heat stress or other inhibitors, such as ethanol, acetic acid, furfural, and 5-HMF, share specific common characteristics, e.g., the accumulation of reactive oxygen species, membrane damage, and the denaturation of macromolecules, which subsequently trigger stress-responsive mechanisms to protect yeast cells from severe stress conditions. Yeast cells that exhibit high resistance to heat may also possess the ability to tolerate other stresses^70^. Thus, all the selected thermotolerant yeast isolates were evaluated for their abilities to withstand acetic acid, furfural, 5-HMF, and ethanol using YM medium supplemented with each inhibitor at different concentrations. As shown in Table 3, the viability of the yeast cells varied depending on the yeast strains and the types of inhibitors. For acetic acid stress, all the selected yeasts, except strains ACP22, LF101, LF119, LFRE2, RBRE2, and MGRE2, grew well at an acetic acid concentration of 80 mM, with cell viability of greater than 50% compared to the control treatment. However, the viability of the yeast cells markedly decreased when the acetic acid concentration increased to 120 mM and 160 mM. At 160 mM acetic acid, only nineteen strains of yeasts exhibited cell survival greater than 20% compared to the control, and most of them belonged to *S. ludwigii*.

**Table 3:** The inhibitory effects of lignocellulosic inhibitors on the relative cell viability (%) of the selected ethanologenic fermentative yeasts.

Yeast isolates	Control	Acetic acid (mM)			Furfural (mM)			5-HMF (mM)			Ethanol (% v/v)		
		80	120	160	5	10	15	10	15	20	4	6	8
ACP20	100	61.23	50.36	25.00	98.62	92.49	64.86	74.32	52.85	42.50	81.08	75.30	54.95
ACP21	100	67.42	54.08	23.97	93.75	80.40	66.15	55.34	49.48	37.11	85.94	83.23	74.48
ACP22	100	46.90	30.53	21.71	70.09	63.24	53.23	56.29	53.42	50.33	86.64	72.85	62.45
ACP24	100	60.30	30.42	20.13	92.95	75.38	57.33	64.74	59.62	49.62	73.08	68.21	62.56
ACP26	100	59.79	51.03	34.54	90.25	80.02	60.25	50.45	45.12	41.87	85.42	79.87	70.96
RR1	100	58.94	57.00	27.05	95.61	90.36	73.69	75.60	70.06	41.09	98.20	92.56	73.09
RR2	100	52.27	47.16	26.15	96.12	91.34	75.23	75.77	71.06	43.09	98.01	95.56	80.42
RR3	100	58.20	30.33	20.20	93.51	90.01	70.30	74.60	70.01	40.02	97.45	90.16	71.73
RR4	100	54.72	33.61	22.41	94.87	90.04	65.45	73.25	69.84	41.09	96.71	91.26	71.31
LF56	100	60.43	32.06	17.43	80.62	60.13	46.71	70.12	50.07	43.27	83.26	71.84	58.70
LF98	100	50.91	29.26	15.37	75.29	51.72	37.32	63.40	41.75	36.70	76.13	67.01	53.19
LF101	100	48.22	26.40	10.65	86.03	65.34	40.28	54.07	48.80	39.42	80.72	69.80	52.83
LF119	100	45.85	24.09	9.03	79.41	57.81	36.12	50.72	44.09	35.81	72.08	64.20	50.17
LF137	100	51.50	37.41	9.77	72.53	51.09	35.40	55.49	47.31	36.50	75.30	62.56	51.80
LFRE1	100	63.95	38.37	22.17	99.50	90.09	72.50	94.11	52.87	42.67	99.86	91.95	80.51
LFRE2	100	47.41	34.66	23.07	98.75	90.16	70.23	87.63	50.11	40.23	98.04	90.85	75.12
SNRM1	100	70.79	59.55	36.52	85.14	82.55	61.30	60.01	54.38	36.32	80.21	76.31	50.36
SNRM2	100	71.80	56.22	34.48	86.25	85.55	64,31	61.01	55.21	36.40	80.78	76.21	51.86
RBRE1	100	52.98	32.26	21.39	97.01	87.39	60.36	70.87	60.05	40.58	74.12	70.34	57.03
RBRE2	100	40.23	25.45	18.35	82.90	67.91	47.08	65.31	40.50	37.40	72.10	68.43	51.62
DGRE1	100	70.25	52.51	29.36	70.45	60.23	50.32	66.78	40.85	40.12	80.33	66.19	54.22
DGRE2	100	80.49	53.16	32.30	72.18	66.14	56.27	67.59	44.62	46.06	83.33	67.19	57.22
APRE1	100	50.03	27.66	14.83	88.41	69.01	47.30	64.09	48.30	40.22	79.81	62.36	50.04
APRE2	100	51.08	39.82	21.33	99.30	98.87	72.16	69.21	55.37	44.07	82.12	67.80	55.37
AC1	100	58.14	28.29	19.38	78.01	59.20	38.39	65.38	43.62	35.21	73.25	63.42	50.45
AC4	100	50.42	23.07	17.31	80.43	61.67	43.54	71.47	50.02	40.06	76.70	66.07	53.78
MGRE1	100	57.02	50.88	25.56	90.25	70.18	65.50	65.32	47.88	43.21	76.89	70.12	56.03
MGRE2	100	48.75	37.50	18.98	88.65	65.41	48.33	60.45	42.31	38.07	74.03	67.42	50.81
OR2	100	65.34	63.28	20.01	91.55	70.03	64.03	60.35	45.84	40.28	75.65	74.23	56.79

Most of the selected yeast isolates exhibited good growth under furfural stress. At the highest concentration of furfural (15 mM), all the yeast isolates, except LF56, LF98, LF101, LF119, LF137, RBRE2, APRE1, AC1, AC4, and MGRE2, displayed cell survival rates higher than 50% compared to the control. Interestingly, *S. ludwigii* strains RR1, RR2, RR3, LFRE1, LFRE2, and APRE2 exhibited greater than 70% cell survival at a furfural concentration of 15 mM compared to other isolates. Regarding the inhibitory effect of 5-HMF at concentrations of 10–20 mM, most of the selected yeast isolates were susceptible to this compound, especially at a concentration of 20 mM. Twenty-three yeast isolates exhibited cell survival greater than 60% at 10 mM 5-HMF, while only fifteen yeast isolates displayed greater than 50% cell survival at 15 mM 5-HMF. Surprisingly, only one yeast isolate, ACP22, exhibited the highest cell survival of 50.33% at the highest concentration of 5-HMF.

Regarding ethanol stress, all the selected yeast isolates can tolerate ethanol concentrations in the range of 4–8% (v/v), although their cell viability was decreased upon an increased ethanol concentration. More than 70%, 60%, and 50% cell survival was detected when the yeast cells were grown in medium supplemented with 4%, 6%, and 8% (v/v) ethanol, respectively. Interestingly, eight yeast isolates, ACP21, ACP26, RR1, RR2, RR3, RR4, LFRE1, and LFRE2, exhibited greater than 70% cell survival in medium supplemented with 8% (v/v) ethanol.

The inhibitory effect of acetic acid, furfural, 5-HMF, and ethanol on the growth of microbial cells has been previously reported. Exposure to weak acids such as acetic acid typically impairs cell growth and alters carbon metabolism. Acetic acid at a low concentration of 60 mM suppresses *S. cerevisiae* growth^71^. In addition, high concentrations of acetic acid negatively impact transmembrane proton transport, affecting pH homeostasis, membrane lipid integrity, and protein stability and eventually causing cell death^71,72^. Aldehyde inhibitors, such as furfural and 5-HMF at a low concentration (0.5–2.0 g/L), are known to cause damage to macromolecules, such as nucleic acids (DNA and RNA), proteins, and cell membranes^70,73^. These aldehyde inhibitors can perturb the metabolic activities of microbial cells either by the direct reaction of the aldehyde functional group or by inducing the accumulation of reactive oxygen species (ROS), which can cause mutation, protein and enzyme inactivation, and cellular damage^74^. Concerning ethanol stress, a high concentration of ethanol negatively affects cell growth by inhibiting cell division and elongation, resulting in a reduction in the specific growth rate and cell viability. Furthermore, high ethanol concentrations also cause the denaturation of macromolecules, such as DNA, RNA, proteins, and enzymes, which may lead to cell death^75,76^.

Based on the inhibitory effect of the lignocellulosic pretreatment inhibitor assay, a total of nineteen yeast isolates exhibited relatively high cell viability under all stress conditions, i.e., greater than 20% cell survival at 160 mM acetic acid, greater than 50% at 15 mM furfural, greater than 35% at 20 mM 5-HMF, and greater than 50% at 8% ethanol, and they were selected for further study. These strains included ACP20, ACP21, ACP22, ACP24, ACP26, RR1, RR2, RR3, RR4, LFRE1, LFRE2, SNRM1, SNRM2, RBRE1, DGRE1, DGRE2, APRE2, MGRE1, and OR2, which belong to *S. ludwigii*. These findings suggest a high possibility of using *S. ludwigii* for high-temperature ethanol production under stress conditions using lignocellulosic biomass as feedstock.

### Effect of acetic acid and furfural on ethanol production by selected fermentative yeasts

The inhibitory effect of acetic acid and furfural, the most predominant lignocellulosic pretreatment inhibitors, on ethanol production by the selected yeast isolates was investigated at 37 °C using YM medium containing 160 g/L glucose and supplemented with either 80 mM acetic acid or 15 mM furfural. The results are summarized in Table 4. The ethanol concentrations and productivities of all yeast isolates under the control condition without inhibitors were higher than those under acetic acid and furfural treatments. The maximum ethanol concentration and productivity of 65.45 g/L and 1.36 g/L.h were achieved by yeast isolate APRE2. Under acetic acid stress, the ethanol concentrations produced by the selected yeasts ranged from 37.83 to 65.08 g/L, with a productivity of 0.79 to 1.36 g/L.h. Nine yeast isolates, i.e., ACP21, ACP26, RR1, LFRE1, LFRE2, RBRE1, DGRE2, APRE2, and MGRE1, displayed ethanol concentrations higher than 50 g/L; among them, the yeast isolate APRE2 had the highest ethanol concentration of 65.08 g/L, while ACP22 gave the lowest value (37.83 g/L). The ethanol production efficiency of the selected yeast isolates was slightly lower under furfural stress than those under the control and acetic acid treatments. The ethanol contents under furfural stress ranged from 26.38 to 47.85 g/L, with a productivity of 0.55 to 1.00 g/L.h. Only four isolates of yeasts, ACP21, LFRE1, DGRE2, and APRE2, exhibited ethanol titers higher than 40 g/L under this stress condition. The yeast isolate APRE2 gave the highest ethanol concentration, while DGRE1 produced the lowest concentration. Both strains belonged to *S. ludwigii*, but their differences in ethanol production performance might be attributed to the differences in genetic background.

**Table 4:** Ethanol production potential of the selected yeasts at 37 °C using YM medium supplemented with 80 mM acetic acid or 15 mM furfural as fermentation medium.

Yeast isolates	Control without inhibitor		80 mM acetic acid		15 mM furfural	
	*P* (g/L)	*Qp* (g/L.h)	*P* (g/L)	*Qp* (g/L.h)	*P* (g/L)	*Qp* (g/L.h)
ACP20	42.11 ± 0.94^j^	0.88 ± 0.02^ k^	41.08 ± 0.23^ m^	0.86 ± 0.01^jk^	33.66 ± 0.49^ h^	0.70 ± 0.03^ fg^
ACP21	56.26 ± 0.92^c^	1.17 ± 0.02^c^	55.71 ± 0.44^d^	1.16 ± 0.02^bcd^	44.38 ± 0.61^b^	0.92 ± 0.02^b^
ACP22	40.54 ± 0.54^ k^	0.84 ± 0.01^ l^	39.83 ± 0.59^n^	0.83 ± 0.02^jk^	38.58 ± 0.45^e^	0.80 ± 0.01^ cd^
ACP24	37.46 ± 0.92^ l^	0.78 ± 0.02^ m^	37.83 ± 0.29^o^	0.79 ± 0.01^ k^	38.54 ± 0.89^e^	0.80 ± 0.04^ cd^
ACP26	55.26 ± 0.35^ cd^	1.15 ± 0.01^ cd^	54.88 ± 0.70^e^	1.14 ± 0.03^ cd^	38.47 ± 0.32^e^	0.81 ± 0.01^ cd^
RR1	52.10 ± 0.69^f.^	1.09 ± 0.01^ g^	50.08 ± 0.27^ h^	1.04 ± 0.01^efg^	34.73 ± 0.65^ g^	0.72 ± 0.02^ef^
RR2	49.21 ± 0.81^ g^	1.03 ± 0.02^ h^	46.38 ± 0.44^j^	0.97 ± 0.02^ghi^	38.58 ± 0.52^e^	0.80 ± 0.01^ cd^
RR3	44.47 ± 0.81^i^	0.93 ± 0.02^j^	42.80 ± 0.50^ l^	0.89 ± 0.03^ij^	33.34 ± 0.57^ h^	0.69 ± 0.02^ fg^
RR4	50.37 ± 0.97^ g^	1.05 ± 0.02^ h^	48.12 ± 0.55^i^	1.00 ± 0.03^fgh^	32.95 ± 0.40^ h^	0.69 ± 0.03^ fg^
LFRE1	61.75 ± 0.73^b^	1.29 ± 0.02^b^	59.17 ± 0.37^b^	1.23 ± 0.02^b^	40.95 ± 0.56^c^	0.85 ± 0.01^c^
LFRE2	53.94 ± 1.03^e^	1.12 ± 0.02^ef^	52.97 ± 0.50^f^	1.10 ± 0.02^de^	33.19 ± 0.44^ h^	0.69 ± 0.03^ fg^
SNRM1	41.26 ± 1.21^jk^	0.86 ± 0.03^kl^	39.91 ± 0.31^n^	0.83 ± 0.01^jk^	37.17 ± 0.59^f^	0.77 ± 0.03^de^
SNRM2	47.82 ± 0.73^ h^	1.00 ± 0.02^i^	46.33 ± 0.40^j^	0.97 ± 0.03^gh^	39.35 ± 0.26^de^	0.82 ± 0.03^ cd^
RBRE1	54.21 ± 0.30^de^	1.13 ± 0.01^de^	53.53 ± 0.66^f^	1.12 ± 0.02^de^	31.97 ± 0.75^i^	0.67 ± 0.03^ fg^
DGRE1	45.55 ± 0.31^i^	0.95 ± 0.01^j^	45.13 ± 0.51^ k^	0.94 ± 0.02^hi^	26.38 ± 0.29^ k^	0.55 ± 0.02^ h^
DGRE2	60.64 ± 0.49^b^	1.26 ± 0.01^b^	58.34 ± 0.70^c^	1.22 ± 0.03^bc^	40.00 ± 0.51^d^	0.83 ± 0.01^ cd^
APRE2	65.45 ± 0.44^a^	1.36 ± 0.01^a^	65.08 ± 0.25^a^	1.36 ± 0.01^a^	47.85 ± 0.60^a^	1.00 ± 0.03^a^
MGRE1	52.64 ± 0.48^f^	1.10 ± 0.01^ fg^	51.89 ± 0.53^ g^	1.08 ± 0.04^def^	31.01 ± 0.24^j^	0.65 ± 0.02^ g^
OR1	50.13 ± 0.37^ g^	1.04 ± 0.01^ h^	47.10 ± 0.02^j^	0.98 ± 0.01^gh^	36.94 ± 0.14^f^	0.77 ± 0.01^de^

*P*, ethanol concentration (g/L); and *Qp*, volumetric ethanol productivity (g/L.h). Mean values ± standard deviation (SD) with different superscript letters in the same column are significantly different at *p* < 0.05 based on DMRT analysis.

Generally, the ability of yeasts to withstand a high level of acetic acid and furfural content depends on several factors, such as the culture medium, fermentation conditions, and yeast species. For instance, the recombinant *S. cerevisiae* strain R32 could tolerate acetic acid and furfural at concentrations of 0.55% (v/v) and 0.3% (v/v), respectively, at 40 °C^77^, while *P. kudriavzevii* strain RGB3.2 isolated from buffalo rumen could withstand 0.4% (v/v) acetic acid and 1.5 g/L furfural at the same temperature^31^. Díaz-Nava et al.^30^ recently reported that the native yeast *P. kudriavzevii* ITV-S42 isolated from sweet sorghum juice could resist acetic acid and furfural at concentrations up to 24 g/L and 1 g/L, respectively, at 40 °C. In the current study, four yeast isolates, ACP21, LFRE1, DGRE2, and APRE2, exhibited relatively high ethanol titers under acetic acid and furfural stress. Thus, they were selected for further evaluation of their ethanol fermentation under multistress conditions at 37 °C.

### Ethanol production efficiency of selected yeasts under multistress conditions using inhibitor cocktail

One of the purposes of the present study is to employ highly efficient selected thermo and acetic acid-tolerant yeasts for ethanol production from acid-treated lignocellulosic hydrolysate, which might contain a mixture of inhibitory substances, especially acetic acid and furfural, the most predominant inhibitory compounds in the lignocellulosic hydrolysate. Therefore, the ethanol production of the selected yeast isolates in the presence of the inhibitor cocktail was determined. A YM medium containing 160 g/L glucose and supplemented with an inhibitor cocktail composed of 80 mM acetic acid and 15 mM furfural at different loadings was used as the fermentation medium to evaluate the ethanol production efficiency of the selected yeasts. As shown in Table 5, all the selected yeast isolates were able to produce ethanol under all the inhibitor cocktails examined here, indicating their tolerance for multistress conditions. The ethanol concentrations and productivities produced by the selected yeasts in the control treatment ranged from 53.52 to 55.38 g/L and 1.12 to 1.15 g/L.h, respectively, while those values, when produced in the presence of the inhibitor cocktails, ranged from 57.10 to 63.07 g/L and 1.19 to 1.31 g/L.h, respectively. The yeast isolate APRE2 gave the highest ethanol concentration of 63.07 g/L, with a productivity of 1.31 g/L.h and yield efficiency of 87.60% in the presence of 50% inhibitor cocktail loading, and 60.02 g/L, with a productivity of 1.25 g/L.h and yield efficiency of 81.58% in the case of 100% inhibitor cocktail loading. Based on this finding, the yeast isolate APRE2 exhibited superior tolerance to multistress conditions; thus, it was chosen for ethanol production using PWH as feedstock in the following experiment.

**Table 5:** Ethanol production potential of the selected yeasts at 37 °C using YM medium supplemented with a cocktail inhibitor containing 80 mM acetic acid and 15 mM furfural.

Yeast isolates	% loading	*P* (g/L)	*Qp* (g/L.h)	*Y* (g/g)	*Ey* (%)
ACP21	N	54.19 ± 0.35^f^	1.13 ± 0.01^f^	0.38 ± 0.01^e^	73.46 ± 0.45^i^
LFRE1	N	53.52 ± 0.14^f^	1.12 ± 0.00^f^	0.37 ± 0.00^e^	72.55 ± 0.19^j^
DGRE2	N	55.14 ± 0.77^e^	1.15 ± 0.02^f^	0.38 ± 0.01^e^	74.80 ± 0.97^ h^
APRE2	N	55.38 ± 0.02^e^	1.15 ± 0.01^f^	0.38 ± 0.01^e^	75.08 ± 0.07^ h^
ACP21	50	62.47 ± 0.50^a^	1.30 ± 0.04^a^	0.43 ± 0.01^b^	84.61 ± 0.20^b^
LFRE1	50	58.70 ± 0.71^c^	1.22 ± 0.01^d^	0.41 ± 0.01^c^	79.49 ± 0.85^f^
DGRE2	50	60.41 ± 0.07^b^	1.26 ± 0.01^b^	0.43 ± 0.01^b^	83.26 ± 0.28^c^
APRE2	50	63.07 ± 0.28^a^	1.31 ± 0.03^a^	0.45 ± 0.01^a^	87.60 ± 0.17^a^
ACP21	100	59.04 ± 0.32^c^	1.23 ± 0.01^ cd^	0.41 ± 0.01^ cd^	80.08 ± 0.94^ef^
LFRE1	100	57.10 ± 0.46^d^	1.19 ± 0.01^e^	0.40 ± 0.00^d^	77.39 ± 0.43^ g^
DGRE2	100	59.23 ± 0.61^c^	1.23 ± 0.02^bcd^	0.41 ± 0.01^ cd^	80.79 ± 0.21^de^
APRE2	100	60.02 ± 0.12^b^	1.25 ± 0.02^bc^	0.42 ± 0.00^bc^	81.58 ± 0.15^d^

*P*, ethanol concentration (g/L); *Qp*, volumetric ethanol productivity (g/L.h); *Y*, ethanol yield (g/g); and *Ey*, ethanol production efficiency (%). Mean values ± standard deviation (SD) with different superscript letters in the same column are significantly different at *p* < 0.05 based on Duncan’s Multiple Range Test (DMRT) analysis.

It should be noted in the current study that the ethanol titers produced in the presence of inhibitor cocktails, especially acetic acid, were slightly higher than those of the control treatment without inhibitors. These findings were similar to those of Taherzadeh et al.^78^ and Thomas et al.^79^, who stated that acetic acid caused a reduction in biomass yield coupled with an increase in ethanol production. This phenomenon can be explained by the diversion of carbon from biomass growth to ethanol production for ATP generation needed to maintain intracellular pH homeostasis and the removal of excess acetate from the cytoplasm through the energy-dependent weak acid efflux pump^80,81^.

### Ethanol production from acid-pretreated PWH by selected yeast

Pineapple is an economic crop with a global production of approximately 24.79 million metric tons annually. Thailand is one of the top countries in pineapple production, producing 2.21 million metric tons, accounting for 8.91% of the world's production^82^. During the pineapple production process, approximately 50% (w/w) of the pineapple weight is discarded as waste, including pineapple peel, core, stem, and leaves^83^. Among these, pineapple peel and core are promising raw materials for second-generation bioethanol production since they are highly biodegradable and rich in proteins and carbohydrates^84^. These pineapple wastes contain a high amount of cellulose (16.57%) and hemicellulose (28.81%) with a low level of lignin (3.04% of the total dry matter)^32^. This study assessed ethanol production from acid-pretreated PWH by the selected yeast *S. ludwigii* APRE2. After a dilute acid pretreatment using 0.5% (v/v) H_2_SO_4_, approximately 106 g/L of total sugars was detected in the PWH, which was markedly higher than that found in the sugarcane bagasse hydrolysate (85 g/L)^29^ and rice straw hydrolysate (34.5 g/L)^31^. Glucose and fructose were the most predominant sugars detected in the PWH, comprising 43.89 and 41.64 g/L, respectively, followed by xylose and arabinose, with concentrations of 5.68 and 4.45 g/L, respectively (Supplementary Fig. S1). Other sugars in pineapple fruit, such as sucrose, were not detected in the PWH. This finding may be explained by the fact that sucrose is converted into glucose and fructose during dilute acid pretreatment, which was similar to that reported by Phong et al.^32^.

**Figure 2:**
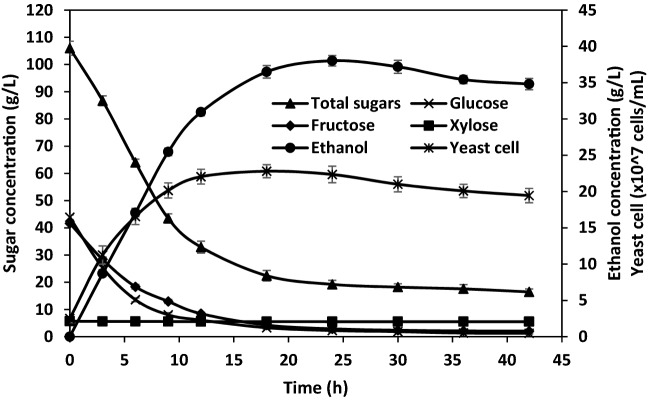
Time profile of ethanol production from pineapple waste hydrolysate using *S. ludwigii* APRE2 at 37 °C.

In addition to fermentable sugars, a dilute acid pretreatment of pineapple waste using 0.5% (v/v) H_2_SO_4_ also liberated the lignocellulosic pretreatment inhibitors, including acetic acid and furfural. No 5-HMF or phenolic compounds were detected in the hydrolysate. The acetic acid and furfural concentrations in the PWH were 131 mM and 3.95 mM, respectively, which were comparable to those reported by Phong et al.^32^. When PWH was directly used as a feedstock for ethanol production at 37 °C using *S. ludwigii* APRE2, a maximum ethanol concentration of 38.02 g/L, the productivity of 1.58 g/L.h, and an ethanol yield of 82.35% were achieved (Fig. 2), suggesting that the growth and ethanol fermentation activity of *S. ludwigii* APRE2 were not affected by the acetic acid and furfural in the raw material. The ethanol production efficiency of *S. ludwigii* APRE2 was compared with other ethanologenic yeasts using different feedstocks, and the results are summarized in Table 6. The ethanol concentration produced by *S. ludwigii* APRE2 was comparable to that produced by *S. cerevisiae* using pomelo peel waste^85^, *S. cerevisiae* TISTR 5048 using pineapple peel waste^86^, *P. kudriavzevii* RZ8-1 using sugarcane bagasse^29^, and *S. cerevisiae* HG1.1 using pineapple waste^32^ as feedstock. In contrast, the ethanol concentration and productivity achieved from *S. ludwigii* APRE2 were approximately 4.1- to 4.7-fold higher than those from *P. kudriavzevii* RGB3.2 and *K. marxianus* RGB4.5, respectively, using rice straw hydrolysate as feedstock^31^. The differences in the ethanol titer may correlate with the initial sugar concentration in the raw material. The rice straw hydrolysate contained 19.10 g/L of total sugar, while the PWH used in this study possessed 106 g/L, which was 5.5-fold higher than that of the rice straw hydrolysate.

**Table 6:** Comparative analysis of ethanol production from PWH using *S. ludwigii* APRE2 and other ethanologenic yeasts using different feedstocks.

Yeast strain	Feedstock	Fermentation parameter					
		T (°C)	S (g/L)	P (g/L)	Qp (g/L.h)	TY (%)	References
*S. cerevisiae*	Pomelo peel waste	30	−	36.00	0.75	73.50	^85^
*S. cerevisiae* TISTR 5048	Pineapple peel waste	30	82.10	27.33	−	65.27	^86^
*P. kudriavzevii* RZ8-1	Sugarcane bagasse	37	85.00	35.51	1.48	81.75	^29^
		40	85.00	33.84	1.41	77.91	
*P. kudriavzevii* RGB3.2	Rice straw	40	19.10	9.32	0.39	95.49	^31^
*K. marxianus* RGB4.5		40	19.10	8.03	0.33	82.27	
*S. cerevisiae* HG1.1	Pineapple waste	40	103.00	36.85	3.07	93.61	^32^
*S. ludwigii* APRE2	Pineapple waste	37	105.65	38.02	1.58	82.35	This study

T (°C), the temperature used for ethanol fermentation; S (g/L), an initial sugar concentration in the raw material; P (g/L), ethanol concentration; Qp (g/L.h), ethanol productivity; and TY (%), theoretical ethanol yield.

Although the newly isolated yeast *S. ludwigii* APRE2 could produce a high ethanol concentration and yield using lignocellulosic biomass as feedstock, it does not ferment xylose and arabinose into ethanol (Fig. 2). Further studies are needed to improve ethanol production efficiency; for instance, exploring the potential of a two-stage fermentation strategy or improving the yeasts’ ability to grow and ferment pentose sugars by adaptive laboratory evolution or genetic engineering strategies. In addition, other yeast strains could be employed to convert xylose into xylitol^87,88^.

## Conclusions

In this study, we successfully isolated twenty-nine thermo and acetic acid-tolerant fermentative yeasts from tropical acidic fruits, comprising three major yeast species, namely *S. ludwigii*, *P. kudriavzevii*, and *P. manshurica*. Of these, *S. ludwigii* APRE2 was the most effective thermo and acetic acid-tolerant yeast for high-temperature ethanol fermentation. It exhibited good growth at temperatures up to 43 °C and could withstand multistress conditions, including acetic acid, furfural, 5-HMF, and ethanol at concentrations up to 160 mM, 15 mM, 20 mM, and 8% (v/v), respectively. The newly isolated yeast, *S. ludwigii* APRE2, produced a maximum ethanol concentration of 63.07 g/L and had a productivity of 1.31 g/L.h in YM medium containing 160 g/L glucose supplemented with a cocktail inhibitor comprising 80 mM acetic acid and 15 mM furfural. It also displayed a high efficiency of ethanol fermentation using PWH containing 131 mM acetic acid and 3.95 mM furfural as a feedstock, with a maximum ethanol concentration and productivity of 38.02 g/L and 1.58 g/L.h, respectively. With further optimization of the fermentation parameters, *S. ludwigii* APRE2 can potentially be used for high-temperature lignocellulosic bioethanol production.

### Submission declaration and verification

Submission of an article implies that the work described has not been published previously in any form.

## Supplementary Information

Supplementary Information.

## Data Availability

The DNA sequence datasets generated during the current study are available in the NCBI repository (https://www.ncbi.nlm.nih.gov/genbank/), with the GenBank accession number shown in Table 1.
