# Supplementary material for: The potential of multistress tolerant yeast, Saccharomycodes ludwigii, for second-generation bioethanol production
Source: Sci Rep. 2022 Dec 21;12:22062. doi: 10.1038/s41598-022-26686-x (PMC9772304; doi:10.1038/s41598-022-26686-x)
Supplement: Supplementary file 1 — Supplementary Information. [file 41598_2022_26686_MOESM1_ESM.docx]

**Supplementary Figure S1.** HPLC profiles of sugars and inhibitors analysis of pineapple waste hydrolysate. (A) the peak of glucose (13.46 m); xylose (14.36 m); and (B) fructose (13.39 m); arabinose (15.26 m); acetic (18.80 m); ethanol (31.21 m).
